# Safety, Pharmacokinetics, and Antiviral Activity of AT-527, a Novel Purine Nucleotide Prodrug, in Hepatitis C Virus-Infected Subjects with or without Cirrhosis

**DOI:** 10.1128/AAC.01201-19

**Published:** 2019-11-21

**Authors:** Elina Berliba, Maxim Bogus, Frédéric Vanhoutte, Pieter-Jan Berghmans, Steven S. Good, Adel Moussa, Keith Pietropaolo, Robert L. Murphy, Xiao-Jian Zhou, Jean-Pierre Sommadossi

**Affiliations:** a ARENSIA Exploratory Medicine, Republican Clinical Hospital, Chisinau, Moldova; b SGS Life Sciences, Antwerp, Belgium; c Atea Pharmaceuticals, Inc., Boston, Massachusetts, USA; d Northwestern University, Chicago, Illinois, USA

**Keywords:** chronic hepatitis C virus infection, pangenotypic, direct-acting antiviral, NS5B, nucleotide, cirrhosis

## Abstract

AT-527 is a novel modified guanosine nucleotide prodrug inhibitor of the hepatitis C virus (HCV) NS5B polymerase, with increased *in vitro* antiviral activity compared to sofosbuvir and a highly differentiated favorable preclinical profile compared to other anti-HCV nucleoside/nucleotide analogs. This was a multiple-part clinical study where multiple ascending doses of AT-527 up to 600 mg (expressed as AT-527 salt form; equivalent to 553 mg free base) once daily for 7 days were evaluated in a randomized, double-blind, placebo-controlled study of treatment-naive, noncirrhotic, genotype 1b, HCV-infected subjects. The highest dose of AT-527 for the same duration was then evaluated in two open-label cohorts of (i) noncirrhotic, genotype 3, HCV-infected subjects and (ii) HCV-infected subjects of any genotype with compensated (Child-Pugh A) cirrhosis. AT-527 was well tolerated for 7 days in all cohorts. At the highest dose tested, mean HCV RNA reductions of up to 2.4 log_10_ IU/ml occurred within the first 24 h of dosing. Mean maximum reductions observed with 7 days of dosing were 4.4, 4.5, and 4.6 log_10_ IU/ml in noncirrhotic subjects with HCV genotype 1b, noncirrhotic subjects with HCV genotype 3, and subjects with compensated cirrhosis, respectively. The systemic half-life of AT-273, the nucleoside metabolite considered a surrogate of intracellular phosphates including the active triphosphate, exceeded 20 h, supporting once-daily dosing. In summary, AT-527 demonstrated rapid, potent, dose/exposure-related, and pangenotypic antiviral activity with similar responses between subjects with and without cirrhosis. Exposure-antiviral response analysis identified 550 mg (free base equivalent) as the optimal dose of AT-527. Safety and antiviral activity data from this study warrant continued clinical development of AT-527 dosed once daily. (This study has been registered in the European Union Drug Regulating Authorities Clinical Trials Database [EudraCT] under number 2017-002148-34 and at ClinicalTrials.gov under identifier NCT03219957.)

## Abstract (precis)

AT-527 is a novel modified guanosine nucleotide prodrug inhibitor of the hepatitis C virus (HCV) NS5B polymerase, with increased *in vitro* antiviral activity compared to sofosbuvir and a highly differentiated favorable preclinical profile compared to other anti-HCV nucleoside/nucleotide analogs.

## INTRODUCTION

There are approximately 71 million people globally who are chronically infected with hepatitis C virus (HCV) (1). The approved all-oral direct-acting antivirals (DAAs) have drastically improved efficacy outcomes with much-improved safety profiles compared to the interferon-containing regimens of the past (2). Sustained virologic response (SVR) rates with these regimens, shown to be presumptive sustained virologic cures, are greater than 90%, with treatment durations reduced to 8 to 12 weeks depending on the regimen and patient population. Despite recent advances, significant challenges remain in managing difficult-to-treat populations, including those with HCV genotype 3 (GT3) infection, cirrhosis, or prior treatment experience (3, 4). While shorter 8-week durations with currently available regimens, such as glecaprevir and pibrentasvir in combination, are effective in noncirrhotic and select cirrhotic populations with compensated cirrhosis, longer treatment durations and more complicated regimens are required in the more difficult-to-treat populations, including patients with advanced liver disease where protease-containing regimens are contraindicated (5, 6). As a class, nucleotide analogs are ideal candidates for inclusion into such DAA regimens for chronic HCV infection since they are potent and pangenotypic and have a high barrier to resistance. To date, sofosbuvir (SOF) remains the only nucleotide analog (uridine) approved globally for the treatment of hepatitis C. Despite high cure rates with SOF-containing regimens, 12 weeks of treatment are needed in most patient populations, with ribavirin still being required in the most compromised patients with decompensated cirrhosis. Current DAA regimens including SOF plus velpatasvir result in numerically lower SVR rates (some <90%) in GT3-infected patients, either treatment experienced or with compensated or decompensated cirrhosis (7–10). An ideal regimen would have high efficacy based on a short treatment duration (8 weeks or less) for all patient populations (including GT3 and cirrhotic patients), similar safety, and less drug-drug interaction potential compared to currently approved regimens. Novel nucleotide analogs which are more potent across all genotypes thus have the potential to shorten treatment durations, improving ease of use and burden on patients regardless of genotype and stage of disease.

**FIG 1:**
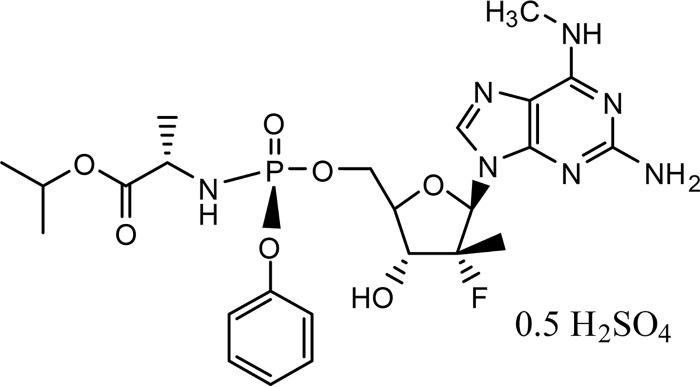
AT-527 chemical structure.

AT-527 (Fig. 1) is a novel liver-targeted nucleotide prodrug for the treatment of chronic HCV infection. AT-527 is a hemisulfate salt of AT-511, a phosphoramidate prodrug of 2′-fluoro-2′-*C*-methylguanosine-5′-triphosphate, a potent and specific inhibitor of HCV viral replication with pangenotypic antiviral activity. The phosphoramidate moiety of the AT-527 structure is identical to that of SOF (11). It is presumed to be subject to the same initial metabolic activation pathway leading to the 5′-monophosphate of 2′-fluoro-2′-*C*-methyl-*N*^6^-methylguanosine, via sequential hydrolysis catalyzed by human cathepsin A (Cat A) and/or carboxylesterase 1 (CES 1) to an l-l-l-alanyl intermediate, followed by removal of the amino acid moiety by histidine triad nucleotide-binding protein 1 (HINT1). The resulting 5′-monophosphate of 2′-fluoro-2′-*C*-methyl-*N*^6^-methylguanosine is then converted to the 5′-monophosphate of 2′-fluoro-2′-*C*-methylguanosine by adenosine deaminase-like protein 1 (ADALP1) and further anabolized to the pharmacologically active 5′-triphosphate (12). The active triphosphate is the predominant intracellular phosphorylated species. Upon dephosphorylation, the nucleoside metabolite 2′-fluoro-2′-*C*-methylguanosine (AT-273) enters the general circulation and is eliminated mostly via the renal pathway (S. S. Good, unpublished data). Since this nucleoside metabolite can be only formed via dephosphorylation of its phosphates, plasma AT-273 is regarded as a surrogate of the intracellular levels of its phosphorylated forms and of the active triphosphate. AT-511, the free base of AT-527, had a 95% effective concentration (EC_95_) of ∼25 nM, being 10-fold more potent than SOF in Huh-7 cells bearing the GT1b replicon (13). AT-511 showed potent antiviral activity *in vitro* against wild-type clinical isolates with EC_95_s less than 80 nM in all HCV genotypes, being ∼10- to 15-fold more potent than SOF in GT1 and GT3 replicons (14). AT-527 has shown no indication of the mitochondrial toxicity observed with prior HCV guanosine nucleotides (14). In addition, animal toxicology studies provided greater than a 200-fold safety margin at the clinical starting dose of 50 mg (expressed as the AT-527 salt form; Good, unpublished).

AT-527 was evaluated in a five-part clinical study. The first two parts included evaluation of sequential, single, escalating doses of AT-527 in healthy subjects (part A) and treatment-naive, noncirrhotic (NC), GT1b HCV-infected subjects (part B). After single oral doses up to 400 mg in healthy subjects and up to 600 mg in HCV-infected subjects, AT-527 was well tolerated. Plasma exposure of AT-273, the nucleoside metabolite of the active nucleotide, was mostly dose proportional with pharmacokinetic (PK) parameters that were comparable in both healthy and HCV-infected subjects. In addition, plasma exposure to AT-273 was similar with or without food. Antiviral activity was dose related, with a mean maximum HCV RNA reduction of 2.3 log_10_ IU/ml after a single 600-mg dose of AT-527 (15, 16).

This work summarizes parts C, D, and E of the study, in which multiple doses of AT-527 were administered (up to 600 mg) for seven consecutive days in HCV-infected subjects with and without cirrhosis. The objectives were to evaluate safety, PK, and antiviral activity after 7 days of AT-527 monotherapy, in order to select the optimal dose for future combination studies.

## RESULTS

### Patient disposition and demographics.

As planned, a total of 36 subjects were enrolled and completed the study with no premature discontinuations or major protocol deviations. Twenty-four subjects participated in part C, 18 receiving active AT-527 and 6 receiving placebo, while 6 subjects who received active AT-527 participated in each of parts D and E.

Subject demographics and baseline characteristics are summarized in Table 1. Compared to subjects without cirrhosis, subjects with cirrhosis (part E) were older with higher baseline FibroScan results. Otherwise, demographics were generally comparable across the dose cohorts, with differences likely due to the relatively small cohort sizes.

**TABLE 1:** Demographics and baseline characteristics^*a*^

Parameter	Part C				Part D, 600 mg/day	Part E, 600 mg/day
	Placebo	150 mg/day	300 mg/day	600 mg/day		
Median age, yr (range)	42 (31–57)	42 (31–64)	38 (31–58)	44 (29–62)	39 (30–44)	56 (39–63)
Median BMI, kg/m^2^ (range)	27.1 (20.1–33.5)	26.0 (20.0–34.6)	24.7 (22.7–26.6)	24.6 (21.3–31.8)	24.7 (21.2–31.8)	26.2 (24.8–34.7)
Female/male, *n* (%)/*n* (%)	3 (50)/3 (50)	1 (17)/5 (83)	3 (50)/3 (50)	4 (67)/2 (33)	0/6 (100)	4 (67)/2 (33)
Race—white, *n* (%)	6 (100)	6 (100)	6 (100)	6 (100)	6 (100)	6 (100)
Median HCV RNA, log_10_ IU/ml (range)	6.2 (5.3–6.9)	5.7 (5.2–6.5)	6.2 (5.4–6.9)	6.2 (5.5–6.5)	6.4 (5.6–7.2)	6.4 (5.7–7.3)
Median ALT, U/liter (range)	48 (28–112)	53 (18–177)	30 (16–47)	50 (10–79)	74 (46–170)	44 (24–63)
Cirrhosis, *n* (%)	0	0	0	0	0	6 (100)
Median FibroScan value, kPa (range)	7.2 (5.6–11.8)	6.6 (3.8–11.1)	6.1 (4.3–6.8)	6.3 (3.3–9.6)	6.8 (4.5–11.0)	17.6 (13.8–31.6)
IL28B status, *n* (%)						
CC	1 (17)	1 (17)	0	2 (33)	4 (67)	1 (17)
CT	2 (33)	5 (83)	4 (67)	3 (50)	1 (17)	4 (67)
TT	2 (33)	0	2 (33)	1 (17)	1 (17)	1 (17)
HCV genotype, *n* (%)						
1b	6 (100)	6 (100)	6 (100)	6 (100)	0	3 (50)
2	0	0	0	0	0	1 (17)
3	0	0	0	0	6 (100)	2 (33)

a All treatments except placebo were AT-527. For all treatment groups, *n* = 6.

### Safety.

No serious adverse events (SAEs), dose-limiting toxicities, or AEs leading to study discontinuation occurred. The most commonly reported nonserious AEs are summarized in Table 2. A higher incidence of lipid-related AEs (mostly low-grade increases in blood cholesterol and blood triglycerides) was observed after administration of AT-527 compared to placebo. There was one asymptomatic, isolated grade 4 elevation of triglycerides which occurred in an NC, GT3-infected subject (part D) 3 days after the last dose of AT-527 and returned to near-normal levels at the next assessment on day 13. This AE was assessed by the investigator as unrelated to study drug. Two subjects with cirrhosis (part E) had AEs of decreased platelet count, but these were assessed as unrelated to study drug as their counts were below the lower limit of normal prior to dosing and were felt to more likely reflect the subjects’ underlying cirrhosis and disease activity. Other than the grade 4 triglyceride elevation, all AEs were mild or moderate in intensity, and most were not attributed to study drug. No other correlation was observed between the incidence, severity, or treatment relatedness of AEs and the administered dose of AT-527. Except for the lipid-related AEs and a trend of decreased alanine aminotransferase (ALT) and aspartate aminotransferase (AST) over time during the treatment period in subjects receiving AT-527 compared to placebo, no clinically relevant or dose-related patterns were observed for any other laboratory parameters, vital signs, or electrocardiogram (ECG) parameters.

**TABLE 2:** Adverse events reported in at least 2 subjects

Adverse event	No. (%) of adverse events^*a*^						
	Part C				Part D, 600 mg/day	Part E, 600 mg/day	Active total, *n* = 30
	Placebo	150 mg/day	300 mg/day	600 mg/day			
Any adverse event	4 (67)	3 (50)	3 (50)	2 (33)	3 (50)	4 (67)	15 (50)
Abdominal pain, upper	1 (17)	1 (17)	1 (17)	0	0	0	2 (7)
Blood cholesterol increased	0	0	1 (17)	2 (33)	2 (33)	1 (17)	6 (20)
Blood glucose increased	0	1 (17)	0	0	1 (17)	0	2 (7)
Blood potassium increased	1 (17)	1 (17)	0	1 (17)	0	0	2 (7)
Blood triglycerides increased	0	1 (17)	2 (33)	1 (17)	1 (17)	1 (17)	6 (20)
Dyspepsia	1 (17)	0	0	0	1 (17)	0	1 (3)
Headache	1 (17)	2 (33)	0	0	0	0	2 (7)
Platelet count decreased	0	0	0	0	0	2 (33)	2 (7)

a All treatments except placebo were AT-527. For all treatment groups except total, *n* = 6.

### Pharmacokinetics.

**FIG 2:**
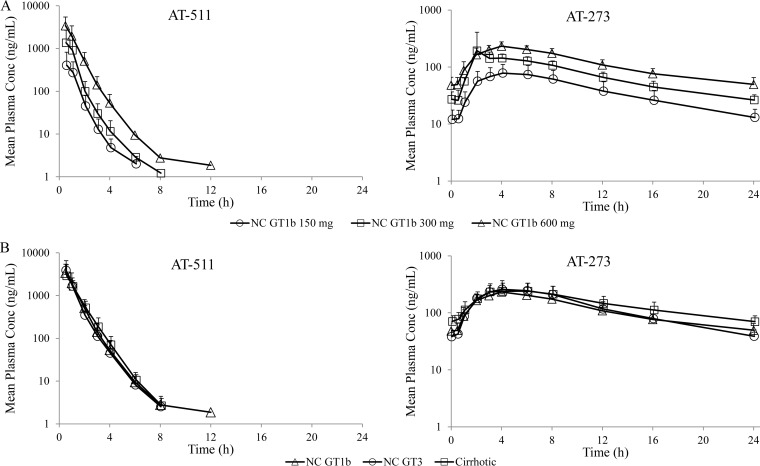
Mean (+SD) steady-state plasma concentration-time profiles of AT-511 and AT-273 on day 7. (A) Ascending doses of 150, 300, and 600 mg QD (6 subjects per dose) in noncirrhotic (NC) GT1b subjects. (B) AT-527 600 mg QD in NC GT1b (*n* = 6), NC GT3 (*n* = 6), and cirrhotic (GT1, -2, or -3; *n* = 6) subjects.

Mean plasma concentration-time profiles are depicted in Fig. 2A for ascending AT-527 doses in subjects without cirrhosis (part C) and Fig. 2B for the 600-mg once-daily (QD) AT-527 dose in subjects with (part E) and without (parts C/D) cirrhosis. Summary plasma PK parameters are presented in Table 3.

**TABLE 3:** Plasma PK parameters of AT-511 and AT-273

Analyte and study part	Dose, mg/day (*n* = 6)	*C*_max_ (ng/ml), mean ± SD		*T*_max_ (h), median (range)		AUC^*b*^ (ng/ml · h), mean ± SD		*T*_1/2_ (h), mean ± SD		*C*_24h_^*c*^ (ng/ml), mean ± SD	
		Day 1	SS^*a*^	Day 1	SS	Day 1	SS	Day 1	SS	Day 1	SS^*c*^
AT-511											
C	150	573 ± 280	462 ± 409	0.5 (0.5–1.0)	1.0 (0.5–1.0)	492 ± 141	475 ± 301	0.62 ± 0.11	0.64 ± 0.20		
	300	2,277 ± 893	1,834 ± 1,313	0.5 (0.5–0.9)	0.5 (0.4–1.0)	1,947 ± 1,120	1,510 ± 976	0.80 ± 0.18	0.73 ± 0.15		
	600	4,211 ± 2,302	3,604 ± 1,742	0.5 (0.5–0.5)	0.5 (0.5–1.0)	3,335 ± 1,502	4,036 ± 2,093	0.86 ± 0.11	0.85 ± 0.12		
D	600	3,971 ± 1,943	4,144 ± 2,280	0.5 (0.5–0.5)	0.5 (0.5–1.0)	3,333 ± 1,241	3,754 ± 2,275	0.73 ± 0.12	0.83 ± 0.06		
E	600	3,412 ± 2,175	3,192 ± 2,085	0.5 (0.5–1.0)	0.5 (0.5–1.0)	3,323 ± 1,467	3,527 ± 1,605	0.86 ± 0.18	0.81 ± 0.12		
AT-273											
C	150	75.6 ± 15.4	81.1 ± 33.9	4.0 (4.0–6.0)	4.0 (4.0–8.0)	800 ± 213	962 ± 409		12.5 ± 6.33	8.08 ± 3.48	12.8 ± 4.45
	300	123 ± 16.6	220 ± 203	4.0 (2.9–6.0)	4.0 (2.0–5.9)	1,414 ± 220	1,828 ± 453		24.5 ± 15.3	18.0 ± 8.83	26.1 ± 7.56
	600	197 ± 57.1	233 ± 42.9	5.0 (4.0–6.0)	4.0 (4.0–6.0)	2,204 ± 486	2,839 ± 572		28.9 ± 14.4	27.5 ± 5.21	46.9 ± 15.5
D	600	195 ± 42.9	263 ± 104	5.0 (3.0–6.0)	4.0 (4.0–6.0)	2,253 ± 595	3,117 ± 1,048		27.9 ± 18.3	30.1 ± 10.9	37.8 ± 11.4
E	600	201 ± 68.1	255 ± 95.4	5.0 (3.0–6.0)	6.0 (4.0–6.0)	2,625 ± 873	3,569 ± 1,214		24.4 ± 9.81	41.6 ± 12.9	69.9 ± 18.5

a SS, steady state.

b AUC_inf_ for AT-511 and AUCτ for AT-273.

c *C*_24_ reported for only AT-273; *C*_24_ at steady state was the mean of *C*_24_ at 72, 96, 120, 144, and 168 h.

Following daily dosing for 7 days in part C, AT-511 exhibited a short half-life and did not accumulate over time. Plasma exposure of AT-511 was slightly more than dose proportional from 150 mg to 300 mg and mostly dose proportional thereafter. While plasma peak and total exposure of AT-273, the guanosine nucleoside metabolite reflecting intracellular active triphosphate, was dose proportional from 150 to 300 mg and less than dose proportional from 300 mg to 600 mg, trough levels of AT-273 were mostly dose proportional in the studied dose range. Based on AT-273 trough levels, steady-state PK was essentially reached after the third or fourth dose. As expected, AT-273 exhibited a long half-life (∼13 to 30 h), which supported QD dosing. The long half-life resulted in the desired higher AT-273 trough (50% to 60%) upon reaching steady state.

As depicted in Fig. 2B and Table 3, the PK of AT-511 and AT-273 were also generally comparable after 600-mg QD AT-527, regardless of HCV genotype infection (part C versus part D) or cirrhosis status (parts C/D versus part E). Steady state was reached after the fifth dose in the cohort of subjects with cirrhosis.

### Antiviral activity.

**FIG 3:**
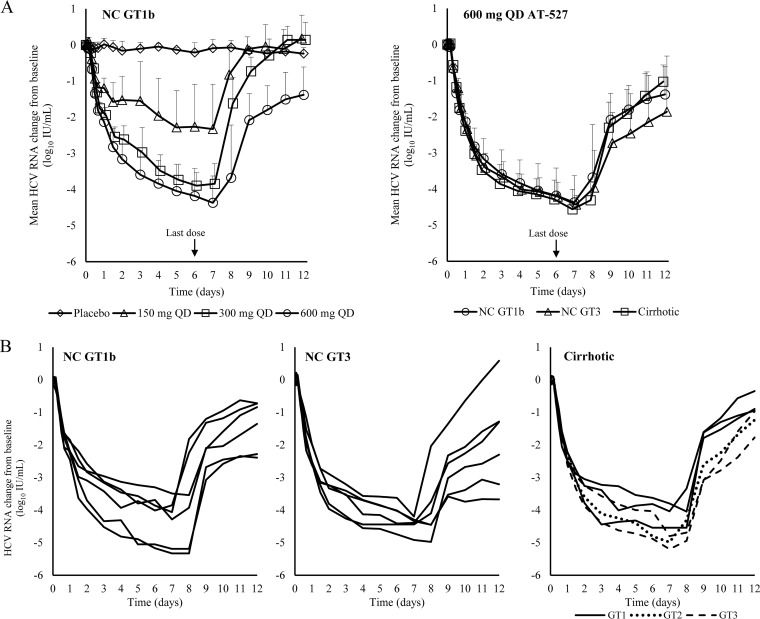
Antiviral activity. (A) Mean (+SD) HCV RNA change from baseline with ascending doses (6 subjects per dose) in noncirrhotic (NC) GT1b subjects and with 600 mg QD in NC GT1b (*n* = 6), NC GT3 (*n* = 6), and cirrhotic (GT1, -2, or -3; *n* = 6) subjects. (B) HCV RNA changes from baseline in individual subjects receiving 600 mg QD.

Dose-related antiviral activity was observed with 7 days of AT-527 dosing in part C, with mean maximum HCV RNA reduction of up to 4.4 log_10_ IU/ml at the 600-mg QD dose in NC GT1b HCV-infected subjects. In parts D and E, the highest dose evaluated in part C (600 mg QD) was also evaluated in NC GT3 and Child-Pugh A (CPA) cirrhotic HCV-infected subjects. In the two populations, similar potent antiviral activities were observed, with mean maximum HCV RNA reduction of 4.5 log_10_ IU/ml in NC GT3 HCV-infected subjects and 4.6 log_10_ IU/ml in cirrhotic HCV-infected subjects. Mean HCV RNA changes from baseline in these populations are presented in Fig. 3A. For comparison, the curves for both the ascending dose cohorts (part C) and all 600-mg QD cohorts (parts C/D/E) are included in the figures.

Individual HCV RNA profiles for all subjects who received 600-mg QD AT-527 in parts C, D, and E are presented in Fig. 3B. Profound early viral response was observed in all subjects after receiving the 600-mg dose, with mean HCV RNA reductions up to 2.4 log_10_ IU/ml within the first 24 h. Five subjects receiving the 600-mg QD dose of AT-527 (3 subjects in part C [50%] and 1 subject each in parts D and E [17%]) achieved HCV RNA levels below the lower limit of quantitation in the study.

**FIG 4:**
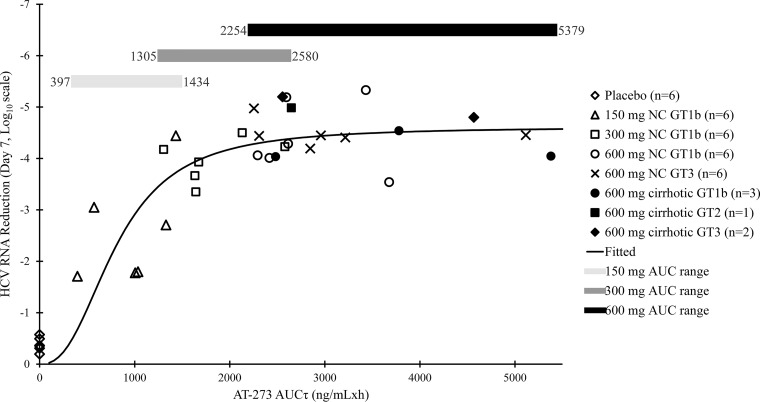
Relationship determined by *E*_max_ modeling between steady-state plasma exposure (AUCτ) of AT-273 and plasma HCV RNA reduction on day 7 from baseline in GT1, -2, or -3 subjects with or without (NC) cirrhosis.

As presented in Fig. 4, the maximum effect (*E*_max_) model described the PK/pharmacodynamics (PD) relationship well. The fitted *E*_max_ curve indicated that maximum viral load reduction of at least 4 log_10_ IU/ml was achieved with the steady-state area under the concentration-time curve (AUCτ) of AT-273 greater than 2,000 ng/ml · h. As displayed in the figure, only the 600-mg QD dose of AT-527 produced AT-273 AUCτ values that were consistently above this threshold.

## DISCUSSION

SOF plus velpatasvir for 12 weeks is the only available nucleoside-containing regimen indicated for all 6 common HCV genotypes. The addition of ribavirin to this regimen is required for patients with decompensated cirrhosis but not for those with compensated cirrhosis. However, emerging data have shown that SOF plus velpatasvir for 12 weeks had poor response (SVR12 = 50%) in patients with HCV GT3b and compensated cirrhosis (10). Similarly low SVR12 rates were also observed in GT3-infected patients with decompensated cirrhosis, despite the doubling of treatment duration to 24 weeks. SVR12 did not improve until ribavirin was added to the regimen (8). These data suggest that SOF may inherently lack potency against HCV GT3, especially in cirrhotic patients. In fact, poor responses in GT3 cirrhotic subjects appeared to be the sole reason for the discontinuation of uprifosbuvir, a uridine nucleotide prodrug structurally close to SOF (17). While contraindicated in patients with decompensated cirrhosis and having a high potential for drug-drug interaction, glecaprevir and pibrentasvir for 8 weeks appeared to be effective in subjects with compensated cirrhosis, although GT3 data are presently lacking (18). Therefore, despite the availability of multiple pangenotypic DAA regimens, there is a continued need for more effective and preferably shorter therapies for HCV, particularly in patients with advanced liver disease (including those with GT3).

AT-527 is a phosphoramidate prodrug of a modified guanosine nucleotide. Past efforts attempting to develop guanosine nucleotide prodrugs such as the cyclic phosphate prodrug PSI-938 were unsuccessful due to clinical toxicities (19). PSI-938 has an O^6^-methyl group rendering its N-glycosylic bond vulnerable to cleavage by cytochrome P450 3A4 (CYP3A4), leading to the formation of a mutagenic metabolite O^6^-methylguanine which is likely responsible for the observed hepatic toxicity of the drug in the clinic (12, 20–23). AT-527 possesses a unique structural feature on its nucleobase, with an N^6^-methyl group that critically differentiates it from these previously developed guanosine nucleotides with respect to metabolic stability. In addition, unlike PSI-938, the activation of AT-527 does not involve CYPs (14). These differences ultimately provide AT-527 with both an enhanced *in vitro* antiviral activity and a favorable preclinical safety profile. Indeed, in *in vitro* studies using clinical isolates, AT-527 was more active than SOF against all genotypes, especially GT3, the most difficult-to-treat genotype, with ∼15-fold-more potency (14). In addition, results from nonclinical studies were fully supportive of initiating clinical development of AT-527.

In the current study, AT-527 was well tolerated up to the highest doses tested (600-mg salt form) for 7 days. The only pattern observed was a higher incidence of mostly low-grade lipid abnormalities (cholesterol and triglyceride increase) in subjects receiving AT-527 compared to placebo. However, this observation is consistent with previously published data showing rapid increase in lipids with HCV clearance upon initiation of DAA therapy in HCV-infected subjects (24, 25). The mechanism is suspected to involve an upregulation of key intrahepatic regulators of lipid metabolism associated with suppression of virus after DAA therapy (24). Thus, the lipid perturbations observed in this study would be expected with the degree of antiviral response achieved in the AT-527-containing cohorts. In addition, there were no other findings suggestive of liver injury. ALT/AST values decreased over time during the treatment period in subjects receiving AT-527, potentially reflecting a decrease in liver inflammation associated with viral load reductions during treatment, an expected effect which will need confirmation with longer-treatment data. Finally, there were no other clinically relevant, dose-related patterns upon analysis of AEs, laboratory parameters, ECGs, and vital signs. Longer-duration studies will be required to confirm the safety of AT-527 beyond 7 days.

After repeated QD administrations for 7 days in a fasted state, AT-511 was quickly absorbed followed by rapid metabolic activation. The formation of AT-273, the guanosine nucleoside metabolite considered a surrogate of intracellular phosphates including the active triphosphate, peaked at approximately 6 h after dosing. Thereafter, AT-273 was slowly eliminated with a long half-life of ∼30.0 h at the 600-mg dose, supporting QD dosing. Steady-state AT-273 concentrations were reached by day 3 or 4 in NC subjects and by day 5 in the subjects with cirrhosis. Overall, mild hepatic impairment did not seem to significantly impact the PK of AT-527 based on plasma exposures of parent drug and metabolites.

Based on the known metabolism of AT-527, the AT-273 metabolite is regarded as the most important metabolite in circulation, as it reflects conversion of AT-527 to the active 5′-triphosphate and subsequent dephosphorylation to its nucleoside metabolite AT-273. AT-273 plasma levels may therefore be a correlate of the antiviral activity of AT-527. For rapidly replicating viruses such as HCV, maintenance of antiviral activity throughout interdose time intervals optimizes efficacy by reducing chances for recrudescent viral replication during interdose trough periods. AT-527 exhibited early and potent viral suppression which correlated well with the plasma PK of AT-273, regardless of genotype or mild hepatic impairment. Upon confirmation of AT-527 antiviral activity in NC GT1b-infected subjects in part C, parts D and E evaluated increasingly more difficult-to-treat NC GT3-infected subjects and subjects with compensated cirrhosis (two of whom also had GT3 infection). After the first 600-mg dose, mean AT-273 trough concentrations (27.5 ng/ml in NC GT1b-infected subjects, 30.1 ng/ml in NC GT3-infected subjects, and 41.6 ng/ml in subjects with cirrhosis) already exceeded the EC_95_ of AT-511 in inhibiting replicons containing HCV constructs of clinical isolates (GT1b EC_95_ of ∼22-ng/ml AT-273 equivalent, GT2 EC_95_ of ∼12 ng/ml, and GT3 EC_95_ of ∼18 ng/ml), resulting in very rapid plasma HCV RNA decreases of up to 2.4 log_10_ IU/ml within the first 24 h of dosing. Upon reaching steady state, AT-273 troughs were 2- to 6-fold EC_95_ values (depending on genotype), exerting sustained suppressive pressure on viral replication, leading to ∼4.5-log_10_-IU/ml reductions in plasma HCV RNA regardless of genotype or cirrhosis status. In those cohorts receiving 600 mg QD, ∼30% of subjects achieved HCV RNA below the lower limit of quantitation with only 7 days of therapy. Further modeling demonstrated that *E*_max_ was achieved with AT-273 AUCτ greater than 2000 ng/ml · h, and only the 600-mg QD dose produced exposure values that were consistently above this threshold. Although there is not a direct SOF monotherapy comparison in these same populations, an approximate mean 4.5-log_10_-IU/ml reduction was observed after 7 days of dosing with SOF in a relatively more nucleoside-responsive population of subjects with GT1a infection and no cirrhosis (26, 27). Thus, considering the limitations of comparison across studies, it is reasonable to conclude that the antiviral kinetics of AT-527 in the clinic is at least comparable to SOF and potentially more profound in the more difficult-to-treat populations, such as those with GT3 infection and cirrhosis. As above, longer-duration combination studies with AT-527 will be needed to further assess viral kinetics and clinical efficacy in such populations. Overall, in this proof-of-concept study, AT-527 monotherapy exhibited very rapid and equally potent antiviral activity regardless of genotype or cirrhosis status.

As noted in Materials and Methods and consistent with the clinical protocol, the doses described in this publication refer to the AT-527 salt form of the drug. Future clinical studies will express the human dose as the AT-511 free base equivalent, consistent with regulatory guidance. As such and based on the results described, a future clinical dose rounded to 550 mg free base (99.5% of the 600-mg salt form [553 mg free base] evaluated in this study) will be selected for future study.

In summary, AT-527 at the highest doses evaluated for 7 days was well tolerated in HCV-infected subjects. AT-527 demonstrated rapid, potent, dose/exposure-related, and pangenotypic antiviral activity with similar responses in those subjects with and without cirrhosis. Although current HCV therapies have generally high SVR rates, treatment durations continue to be at least 12 weeks for many populations with some regimens that are either contraindicated or still requiring ribavirin in the most compromised decompensated patient population. Considering the PK/PD profile, AT-527 is an ideal candidate for combination with another HCV DAA (such as an NS5A inhibitor) in later-phase studies, with a goal of potentially shortening treatment duration (8 weeks or less) in broad HCV patient populations, including those with GT3 infection and/or cirrhosis. Indeed, *in vitro* studies demonstrated additivity to synergistic effects when AT-527 was combined with an NS5A inhibitor (Good, unpublished). The safety, PK, and antiviral activity data from this proof-of-concept study support a 550-mg free base dose of AT-527 in future phase 2 clinical studies.

## MATERIALS AND METHODS

### Study design.

Part C was a randomized, double-blind, placebo-controlled, multiple ascending dose study. Three dosing cohorts (150 mg, 300 mg, and 600 mg) were evaluated in which eight subjects within each cohort were randomly assigned to receive active AT-527 (*n* = 6) or placebo (*n* = 2) once daily (QD) for 7 days. Dosing of each sequential cohort occurred only after review of safety and PK data of the prior cohort by the established Safety Monitoring Committee. Part C was conducted in 24 treatment-naive, NC, genotype 1 HCV-infected subjects.

Parts D and E were open-label multiple-dose studies in which six subjects in each part were assigned to receive 600 mg AT-527 QD for 7 days. Enrollment in parts D and E initiated only after review of data from part C by the established Safety Monitoring Committee. Part D was conducted in 6 treatment-naive, NC, genotype 3 HCV-infected subjects, and part E was conducted in 6 treatment-naive subjects with compensated (Child-Pugh A [CPA]) cirrhosis. HCV-infected subjects with GT1, -2, -3, -4, -5, or -6 were eligible for part E.

Treatment in part C was assigned via a computer-generated randomization list, and the investigator, research staff, and subjects were blind to treatment for the entire study. AT-527 or matching placebo (if applicable) tablets were administered under fasting conditions. The doses refer to the AT-527 salt form. Subjects were required to stay confined to the clinic facility from the day before dosing (day −1) until clinic discharge on day 13. An outpatient follow-up visit for safety was conducted 3 weeks after clinic discharge.

The study was performed in accordance with the Declaration of Helsinki, International Clinical Harmonization guidelines, Good Clinical Practice, and applicable regulatory requirements. Independent Ethics Committee approval to conduct the study was obtained before enrollment of subjects. Informed consent was obtained from individual subjects before any study-specific procedures were conducted. Subjects from parts C, D, and E were enrolled at a single center in Moldova. The first subject was enrolled in October 2017, and the last subject completed the study in June 2018.

### Patients.

Subjects enrolled in the study were male or female adults, 18 to 65 years of age, with a body mass index (BMI) of 18 to 35 kg/m^2^. Subjects had documented clinical history compatible with chronic HCV, which was defined as any one of the following: (i) positive anti-HCV antibody, (ii) HCV genotyping results, (iii) HCV RNA detected in plasma, and (iv) histological evidence of infection. Serological confirmation of chronicity was available from tests conducted at least 6 months prior to screening. All subjects were treatment naive, with plasma HCV RNA levels of ≥5 log_10_ IU/ml at screening. Part C and part D subjects had GT1 and GT3 infection, respectively, while part E allowed subjects with any genotype. Subjects in parts C and D were noncirrhotic, while subjects in part E had compensated CPA cirrhosis. All subjects had transient elastography (FibroScan) at screening to document either the absence (≤12.5 kPa; parts C and D) or presence (>12.5 kPa; part E) of cirrhosis. In addition, all agreed to use a double method of birth control from screening through 90 days after the last dose of study drug.

Subjects who had any of the following criteria were excluded: pregnancy, breastfeeding, HBV or HIV coinfection, active alcohol or drug abuse, decompensated liver disease, history or findings suggestive of hepatocellular carcinoma, primary or secondary causes of other liver diseases, poorly controlled diabetes mellitus, ALT/AST >5× upper limit of normal (ULN), platelet count <120 × 10^9^/liter (or <60 × 10^9^/liter for subjects with cirrhosis), clinically relevant electrocardiogram (ECG) abnormality, estimated glomerular filtration rate of <60 ml/min/1.73 m^2^, or any other clinically significant medical condition or laboratory abnormality that would jeopardize subject safety or study validity.

### Assessments.

Safety was evaluated through review of adverse events (AEs), physical examination, and assessment of ECGs, vital signs, and clinical laboratory tests (hematology, coagulation, biochemistry, and urinalysis). Concomitant medications were also monitored. Investigators assessed each AE for severity and relationship to study drug.

Serial blood samples were collected for PK analyses over 24 h on day 1 and over 120 h after the last dose on day 7, with predose trough samples collected on days 2 through 6. Plasma concentrations of AT-511 (free base of AT-527), and the guanosine metabolite AT-273 were measured using a validated liquid chromatographic method with mass spectrometric detection (liquid chromatography-tandem mass spectrometry [LC-MS/MS]). Briefly, to 100 μl of plasma samples (samples from subjects, blank, 11 nonzero standards ranging from 1 to 1,000 ng/ml, and 4 quality control samples ranging from 1 to 750 ng/ml) was added 25 μl of each of the internal standard solutions at 400 ng/ml (deuterated AT-511 and deuterated AT-273). To the mixture was then added 300 μl of cold methanol-acetonitrile (3:1, vol/vol). After vortex mixing, protein was removed by centrifugation, and a 50-μl aliquot of the supernatant was transferred to a 96-well plate already containing 400 μl of a mixture of water, methanol, and acetonitrile (90%:7.5%:25%, vol/vol/vol). Fractions of 4 to 8 μl were injected onto the liquid chromatographic system. Reverse-phase chromatography was carried out using an Xbridge C_18_ column (50 by 4.6-mm inside diameter [i.d.], 3.5 μm; Waters Cooperation, Milford, MA) with a linear gradient elution of 98% mobile phase A (10 mM ammonium bicarbonate solution) and 2% mobile phase B (methanol-acetonitrile, 1:1, vol/vol) at time zero to 2% A and 98% B at 3 min at a flow rate of 1 ml/min. Under these conditions, the retention times of AT-511 and AT-273 were 3.35 and 1.90 min, respectively. Eluent was directed to a triple quadrupole mass analyzer. AT-511, AT-273, and their deuterated internal standards were monitored at mass transition 582.22 to 376.20 (AT-511), 585.24 to 379.20 (deuterated AT-511), 300.11 to 152.20 (AT-273), and 303.13 to 152.20 (deuterated AT-273). The lower and upper limits of quantification were 1.00 and 1000 ng/ml for each analyte, respectively. Intra- and interassay accuracy and precision were 96.3 to 107.0% and 1.6 to 9.2%, respectively. Plasma PK parameters, including area under the plasma concentration-time curve (AUC), maximum concentration (*C*_max_), time to *C*_max_ (*T*_max_), predose trough concentration (*C*_24h_), and observed half-life (*T*_1/2_), were estimated using a noncompartmental approach.

Similarly, serial blood samples were also collected for quantitation of HCV RNA on day −1, day 1 (predose and 2, 4, 8, 12, and 16 h postdose), day 2 (predose and 12 h postdose), days 3 to 6 (predose), day 7 (predose and 24, 48, 72, 96, and 120 h postdose), day 13, and day 35. Plasma HCV RNA determinations were performed through use of a validated commercial assay, Cobas AmpliPrep/TaqMan HCV test v2.0, with a limit of quantitation (LOQ) of 15 IU/ml.

### Statistical analysis.

All subjects who received at least one dose of AT-527 or placebo were included in the safety analysis set. AEs were coded using the Medical Dictionary for Regulatory Activities (MedDRA) version 20.0 and summarized by number and percentage of subjects experiencing AEs. All subjects who received AT-527 with evaluable data were included in the PK analysis set. PK parameters were summarized by dose cohort. All subjects who received AT-527 or placebo with evaluable data were included in the pharmacodynamic (PD) analysis set. Log-transformed HCV RNA concentrations at each time point and maximum measured effects were summarized descriptively. Baseline for the PD analyses was considered the mean of the day −1 and the day 1 predose HCV RNA values. For calculations of HCV RNA change from baseline, 15 IU/ml was used for any value that was below LOQ.

As AT-527 exhibited similar anti-HCV activity independently of HCV genotype and cirrhotic status, HCV RNA reductions from baseline after 7 days of QD dosing from part C (150 to 600 mg in GT1), part D (600 mg in GT3), and part E (600 mg in cirrhotic subjects) were pooled to perform exposure (PK)-antiviral activity (PD) analysis. The steady-state AUC of AT-273 (AUCτ) was selected as the PK parameter reflecting total exposure for the PK/PD analysis. A standard *E*_max_ model was used. Model parameters included *E*_max_ (the maximum reduction in HCV RNA from baseline), EC_50_ (AT-273 AUCτ resulting in 50% *E*_max_), and the Hill factor. *E*_max_ analysis was performed using WinNonlin. Statistical analyses were performed using SAS v9.2 or higher (SAS Institute, Cary, NC, USA).
